# Impaired Adaptive Motor Learning Is Correlated With Cerebellar Hemispheric Gray Matter Atrophy in Spinocerebellar Ataxia Patients: A Voxel-Based Morphometry Study

**DOI:** 10.3389/fneur.2019.01183

**Published:** 2019-11-14

**Authors:** Kyota Bando, Takeru Honda, Kinya Ishikawa, Yuji Takahashi, Hidehiro Mizusawa, Takashi Hanakawa

**Affiliations:** ^1^Department of Advanced Neuroimaging, Integrative Brain Imaging Center, National Center of Neurology and Psychiatry, Tokyo, Japan; ^2^Department of NCNP Brain Physiology and Pathology, Graduate School, Tokyo Medical and Dental University, Tokyo, Japan; ^3^National Center Hospital, National Center of Neurology and Psychiatry, Kodaira, Japan; ^4^Motor Disorders Project, Tokyo Metropolitan Institute of Medical Science, Tokyo, Japan; ^5^Department of Neurology and Neurological Science, Graduate School, Tokyo Medical and Dental University, Tokyo, Japan; ^6^Department of Integrated Neuroanatomy and Neuroimaging, Kyoto University Graduate School of Medicine, Kyoto, Japan

**Keywords:** voxel-based morphometry, spinocerebellar degeneration, spinocerebellar ataxia, prism adaptation, MRI, volumetric MRI, motor learning

## Abstract

**Objective:** To evaluate the degree to which recently proposed parameters measured via a prism adaptation task are correlated with changes in cerebellar structure, specifically gray matter volume (GMV), in patients with spinocerebellar degeneration (SCD).

**Methods:** We performed whole-brain voxel-based morphometry (VBM) analysis on 3-dimensional T1-weighted images obtained from 23 patients with SCD [Spinocerebellar ataxia type 6 (SCA6), 31 (SCA31), 3/Machado-Joseph disease (SCA3/MJD), and sporadic cortical cerebellar atrophy (CCA)] and 21 sex- and age-matched healthy controls (HC group). We quantified a composite index representing adaptive motor learning abilities in a hand-reaching task with prism adaptation. After controlling for age, sex, and total intracranial volume, we analyzed group-wise differences in GMV and regional GMV correlations with the adaptive learning index.

**Results:** Compared with the HC group, the SCD group showed reduced adaptive learning abilities and smaller GMV widely in the lobules IV-VIII in the bilateral cerebellar hemispheres. In the SCD group, the adaptive learning index was correlated with cerebellar hemispheric atrophy in the right lobule VI, the left Crus I. Additionally, GMV of the left supramarginal gyrus showed a correlation with the adaptive learning index in the SCD group, while the supramarginal region did not accompany reduction of GMV.

**Conclusions:** This study indicated that a composite index derived from a prism adaptation task was correlated with GMV of the lateral cerebellum and the supramarginal gyrus in patients with SCD. This study should contribute to the development of objective biomarkers for disease severity and progression in SCD.

## Introduction

Spinocerebellar degeneration (SCD) is a degenerative neurological disease that manifests as brain atrophy in multiple brain areas, particularly the cerebellum, and is characterized by clinical manifestations of ataxic gait, dysarthria, dysmetria, and dysdiadochokinesia (1). To date, more than 40 subtypes of SCDs and 38 causative genes are identified (2). SCD is roughly classified into a pure cerebellar atrophy type (e.g., SCA6 and SCA31) and a multiple system atrophy type (e.g., SCA1, 2, and 3) (1).

Previous interventional and cross-sectional studies of SCD have mostly adopted omnibus functional motility scores such as the Scale for the Assessment and Rating of Ataxia (SARA) as outcome measure (3, 4). To advance the development of therapeutic options, development of a fine-graded, specific, quantitative measure for the assessment of cerebellar function is necessary, particularly one that is applicable even at the early stages of the disease.

The cerebellum has multiple functions in motor regulation, learning, and behavior (5, 6). Since the pioneering theoretical work of Albus (7) and Marr (8), the cerebellum has been considered a crucial brain structure for motor learning through the development of “internal models (9–11)”. Indeed, experimental evidence supports the contribution of the cerebellum to conditioning (12), habituation (13), and scaling of various reflexes (14, 15). The cerebellum has also been implicated in complex forms of motor learning, such as sensorimotor adaptation to visual and mechanical distortions. Indeed, previous clinical studies observed that sensorimotor adaptation was often reduced or abolished in SCD patients (16, 17). Among existing sensorimotor adaptation tasks, prism adaptation has been long known as a method for testing cerebellar function and has been used to assess sensorimotor adaptation in individuals with SCD (18, 19).

However, which quantitative measures from prism adaptation are useful in the assessment of cerebellar functions in SCD patients is unclear. Recently, Hashimoto et al. developed the Adaptability Index (*AI*), which is a composite index computed from several parameters measured during prism adaptation, and demonstrated its clinical efficacy in discriminating patients with SCD from healthy people (20) However, whether the *AI* is associated with the degree of cerebellar atrophy and which sectors of the cerebellum are responsible for reduced *AI* values in SCD patients remain unclear.

Voxel-based morphometry (VBM) is a well-established image analysis technique that provides an unbiased and comprehensive assessment of anatomical differences throughout the brain with high local specificity (21) VBM analysis is regarded as a useful method for elucidating the pattern of brain atrophy across different types of SCD and for correlating disease severity with the degree of brain atrophy (22–25).

Here, we conducted structural magnetic resonance imaging (MRI) and compared gray matter volume (GMV) with scores on a hand-reaching task with prism adaptation in patients with SCD and healthy controls (HC). Our objective was to examine the relationship between adaptive learning ability and regional patterns of cerebellar atrophy.

## Materials and Methods

### Participants

Twenty-three patients with SCD were enrolled (Table 1). In the SCD group, 22 patients had genetically confirmed spinocerebellar ataxia (SCA) (26–28) and the remaining one patient was diagnosed clinically as CCA (29, 30). All of the patients were assessed using the SARA (31). The disease onset was defined as the age at which the patients first noticed signs of cerebellar ataxia. This allowed us to define disease duration. As an HC group, 21 age- and sex-matched healthy people were recruited (Table 2). All participants were right-handed. The results of neurological examinations were normal for the individuals in the HC group, indicating no subjective/objective cognitive disturbances. In both the HC and SCD groups, the exclusion criteria were a Mini Mental State Examination (MMSE) score of <24 point (32) and organic brain lesions as indicated via MRI, including age-related changes in white matter (a periventricular hyperintensity rating of 2 or greater or a deep white matter hyperintensity rating of 2 or greater) (33). The SCD group and HC group were similar in terms of mean age [SCD group = 61.3 ± 10.1 years old and HC group = 57.8 ± 11.1 years old; *t*_(42)_ = 1.1, *p* = 0.27], sex distribution (13 males and 10 females in SCD group; 8 males, and 13 females in HC group = *x*^2^ = 1.49, *p* = 0.22), and MMSE score [SCD group = 28.3 ± 2.2, HC group = 28.8 ± 1.4, *t*_(42)_ = 0.96, *p* = 0.34]. This study was carried out in accordance with the recommendations of Bioethics Committee of National Center of Neurology and Psychiatry. All subjects gave written informed consent in accordance with the Declaration of Helsinki. The protocol was approved by the Bioethics Committee of National Center of Neurology and Psychiatry.

**Table 1:** Characteristics of the SCD.

**ID**	**Diagnosis**	**Duration**	**SARA**	**MMSE**	**AI**	****τ**_log**	**TIV**
SCD01	SCA31	204	10	30	0.15	4.9	1.5
SCD02	SCA31	108	17	26	0	2.5	1.51
SCD03	SCA31	132	8.5	30	0.26	15	1.58
SCD04	SCA31	204	1	30	0.6	2.8	1.59
SCD05	SCA31	36	6.5	30	0.14	1.2	1.46
SCD06	SCA31	132	9	30	0.14	4	1.39
SCD07	SCA31	60	8	29	0.36	2.3	1.71
SCD08	SCA31	120	7.5	30	0.58	1.7	1.4
SCD09	SCA31	96	7	25	0.81	15.3	1.32
SCD10	SCA31	144	16	30	0.29	15.7	1.74
SCD11	SCA6	96	12	30	0.25	3.5	1.46
SCD12	SCA6	108	12.5	26	0.16	2.9	1.27
SCD13	SCA6	60	8.5	30	0.2	2.5	1.58
SCD14	SCA6	120	13.5	28	0	3.2	1.36
SCD15	SCA6	120	17.5	24	0.08	0.6	1.44
SCD16	SCA6	60	8	30	0.65	3.3	1.56
SCD17	SCA6	108	14	25	0	14.7	1.54
SCD18	SCA3/MJD	120	9	30	0.24	3	1.4
SCD19	SCA3/MJD	84	15.5	25	0.1	3.8	1.48
SCD20	SCA3/MJD	84	18.5	30	0.19	4.9	1.51
SCD21	SCA3/MJD	60	15.5	28	0.11	1.7	1.43
SCD22	SCA3/MJD	108	13	25	0.18	2.1	1.45
SCD23	CCA	24	11.5	29	0.08	13.9	1.47
Mean ± SD (SCD)		103.8 ± 43.7	11.3 ± 4.2	28.2 ± 2.2	0.24 ± 0.21	5.5 ± 5.1	1.48 ± 0.11
Mean ± SD (HC)		NA	NA	28.8 ± 1.4	0.71 ± 0.18^*^	1.4 ± 1.1^*^	1.47 ± 0.18

*SCD, patients with spinocerebellar degeneration; SCA31, 6, spinocerebellar ataxia type 31, 6; SCA3/NJD, Machado-Joseph disease; CCA, cortical cerebellar atrophy; HC, healthy control; Duration, month of disease duration; SARA, Scale for the Assessment and Rating of Ataxia; MMSE, Mini-Mental State Examination; AI, adaptability index; τ_log, time constant after the natural logarithmic conversion; TIV, total intracranial volume; NA, not available; SD, standard deviation*.

* *p <0.05*.

**Table 2:** Characteristics of the HC group.

**ID**	**MMSE**	**AI**	****τ**_log**	**TIV**
HC01	29	0.8	2.17	1.370
HC02	28	0.28	2.05	1.186
HC03	30	0.6	2.77	1.173
HC04	30	1	1.92	1.728
HC05	28	0.54	−0.16	1.404
HC06	28	0.8	1.43	1.363
HC07	30	0.9	2.04	1.514
HC08	28	0.72	0.15	1.310
HC09	30	0.8	1.21	1.334
HC10	28	0.8	1.56	1.697
HC11	29	1	1.42	1.331
HC12	30	0.72	2.28	1.472
HC13	29	0.576	1.71	1.611
HC14	29	0.81	2.55	1.380
HC15	28	0.288	1.18	1.192
HC16	27	0.72	−2.43	1.520
HC17	30	0.8	0.57	1.825
HC18	28	0.72	2.41	1.551
HC19	29	0.64	2.17	1.632
HC20	28	0.8	2.02	1.628
HC21	28	0.6	0.84	1.597
Mean ± SD	28.8 ± 1.4	0.71 ± 0.18	1.4 ± 1.1	1.47 ± 0.18

*HC, healthy control; MMSE, Mini-Mental State Examination; AI, adaptability index; τ_log, time constant after the natural logarithmic conversion; TIV, total intracranial volume; SD, standard deviation*.

### Prism Adaptation Experiment

The experimental apparatus was previously described by Hashimoto et al. (20). In brief, the participants wore goggles containing a Fresnel prism plate, which shifted the field of view 25° to the right. An electrically controlled shutter screen was installed in the goggles to interrupt visual information during reaching. A participant initially touched his/her index finger to a sensor switch attached to the right earlobe, and then a target (a white circle with a 15-mm radius) was presented on a touchscreen. When the participant started a reaching movement, the release of the sensor switch triggered the electrical shutter, which prevented the participant from seeing his/her hand and the target during reaching. The participant was able to see the target and their hand only after the screen had been touched and the electrical shutter had reopened. In the next trial, the participant attempted to correct his/her reaching movements on the basis of the reaching error in the previous trial. We used original software (Katano Tool Software) on a personal computer to control the task.

The participants performed 100 practice trials without the prism lens, followed by three consecutive test sessions: (1) 50 trials without the prism plate (BASELINE), (2) 100 trials with the prism plate (PRISM), and (3) 50 trials without the prism plate (REMOVAL). According to previous research (20), trials with a correct touch movement were defined as those with a touch error below 25 mm.

### Analysis of the Prism Experiment Data

We analyzed the data from the prism adaptation trial using the method proposed by Hashimoto et al. (20). First, we calculated *AI* as follows:

$$\begin{array}{l} AI=a\;\times b\times c \end{array}$$

where “*a*” is the adaptation index defined as the probability of correct touches in the last 10 trials of the PRISM session; “*b*” is the retention index defined as the probability of incorrect touches in the initial five trials of the REMOVAL session; and “*c*” is the extinction index designated as the probability of correct touches in the last 10 trials of the REMOVAL session. The AI score is a composite index that primarily reflects the combined ability for motor adaption including initial adaptation, retention, and re-adaptation.

Second, we measured the magnitude of finger touch errors (*r*) as the degree of horizontal displacement between the touch points and the center of the target on the touchscreen. The adaptation curve in PRISM session was fitted as follows:

$$\begin{array}{l} \text{r}=\eta \cdot \text{exp}\left(-\text{t}/\tau \right) \end{array}$$

where “η” is the degree of finger touch errors in the first trial of the PRISM session; “t” is the trial number from the start of the session; and the time constant “τ” is the number of trials that had been completed in a session at which the finger touch error approached 36.8% of η. Finally, we performed natural logarithmic conversion according to the skewed distribution of τ (τ*_log*).

### MRI Acquisition

MRI measurements were performed using a 3-Tesla scanner (Siemens Verio) with a 32-channel phased array head coil. Structural MRI was acquired with a 3-dimensional T1-weighted magnetization-prepared rapid gradient echo (MPRAGE) sequence: sagittal acquisition, repetition time (TR) = 1,900 ms, echo time (TE) = 2.52 ms, inversion time (TI) = 900 ms, flip-angle (FA) = 9°, field of view (FOV) = 256 mm, 256 × 256 acquisition matrix, and a voxel size of 1 × 1 × 1 mm^3^. We also referred to a T2-weighted sequence (TR = 4,000 ms, TE = 91 ms, FA = 150°, FOV = 220, 256 × 256 matrix, a voxel size of 0.5 × 0.5 × 3.0 mm^3^, and a 10 mm gap between slices).

### Voxel-Based Morphometry With DARTEL

The MRI data were processed using statistical parametric mapping 12 (SPM12; Wellcome Trust Centre for Neuroimaging) in MATLAB version 9.1.0 (Mathworks). For each participant, the T1-weighted image was segmented into gray matter (GM), white matter (WM), cerebrospinal fluid (CSF), skull, and scalp using the “new segment” routine. Then, the GM images were rigid body-aligned to the tissue probability maps in Montreal Neurological Institute (MNI) standard space. We created a study-specific template by averaging images from 23 patients with SCD and 21 subjects from the HC group, using the DARTEL (diffeomorphic anatomical registration through exponentiated Lie algebra) toolbox (34). Subsequently, all native-space GM images were registered to this study-specific template and further spatially normalized into standard MNI space (1.5 mm isotropic voxel). The resulting GM images were modulated using the Jacobian determinant of the corresponding deformation, filed to correct for volume changes. Finally, the modulated GM images were smoothed using an isotropic Gaussian kernel of 8 mm full-width at half maximum.

### Statistical Analysis

All statistical analyses of demographic data were performed using R version 3.3.3. (35). Age, sex, MMSE scores, and adaptive learning ability parameters (*AI* and τ*_log*) were compared between the groups using two-sample Student's *t*-tests and Pearson's chi-squared test. Spearmans's rank order correlation (r_s_) was used to examine the strength of the association between two variables: between *AI* and SARA score, between *AI* and disease duration, between τ*_log* and SARA score, and between τ*_log* and disease duration. All data are reported as the mean ± standard deviation (SD). The *P*-value for statistical significance was set at < 0.05. To examine between-group differences in regional GMV, we used a voxel-wise general linear model with a 1-factor 2-level ANCOVA design with age, sex, and TIV as covariates (36). To examine correlations between regional GMV and adaptive learning ability parameters, we performed a multiple regression analysis after controlling for age and TIV. A peak-level threshold was initially set at *P* < 0.05 with family-wise error (FWE) correction for multiple comparisons. This conservative approach assumed significant relationship between GMV and the effects of interest in a voxel-wise manner. We also used a second, complementary approach in which we assumed moderate relationship between GMV and effects of interest might be expanded in space beyond the chance level. For this cluster-level approach, the initial voxel-wise peak threshold was set at *P* < 0.001 uncorrected, and the resultant clusters were considered significant when falling below an extent threshold of FWE-corrected *P* < 0.05. For this approach, since structural images display local variation in smoothness, cluster-level correction was applied using Random Field Theory combined with non-stationary correction (37).

## Results

### Differences in Adaptive Learning Ability Parameters Between Groups

*AI* was significantly lower in the SCD group compared with the HC group [SCD group = 0.24 ± 0.22, HC group = 0.71 ± 0.18; *t*_(42)_ = 7.57, *p* < 0.001]. Also, τ*_log* was significantly larger in the SCD group compared with the HC group [SCD group = 5.45 ± 5.09, HC group = 1.42 ± 1.15; *t*_(42)_ = 3.47, *p* < 0.001].

### Correlation of Adaptive Learning Ability Parameters With Clinical Indices of SCD

**Figure 1:**
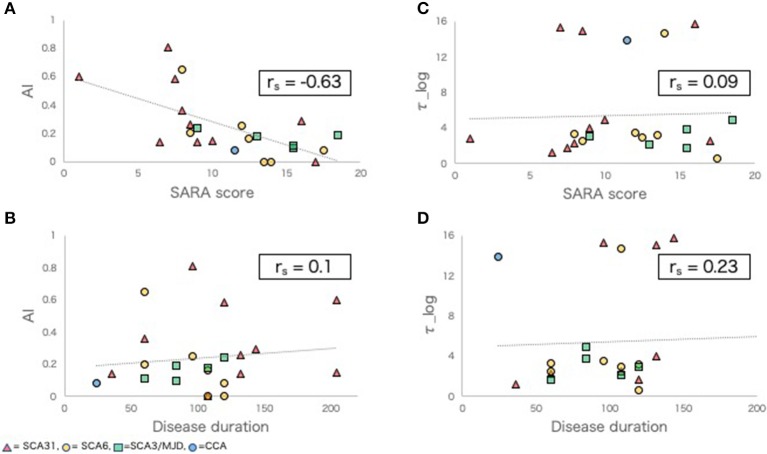
Adaptive learning indexes and other clinical indexes in SCD group. **(A,B)** Scatter plots comparing *AI* with SARA score **(A)**, disease duration **(B)**. **(C,D)** Scatter plots comparing τ*_log* with SARA score **(C)**, disease duration **(D)**. r_s_, Spearman's rank order correlation coefficients.

In the SCD group, a negative correlation was observed between *AI* and SARA score (r_s_ = −0.63, *p* < 0.01, Figure 1A). There was no significant correlation between *AI* and disease duration (r_s_ = 0.1, *p* = 0.66, Figure 1B), between τ*_log* and SARA (r_s_ = 0.09, *p* = 0.69, Figure 1C), or between τ*_log* and disease duration (r_s_ = 0.23, *p* = 0.32, Figure 1D).

### Differences in Regional GMV Between Groups

**Figure 2:**
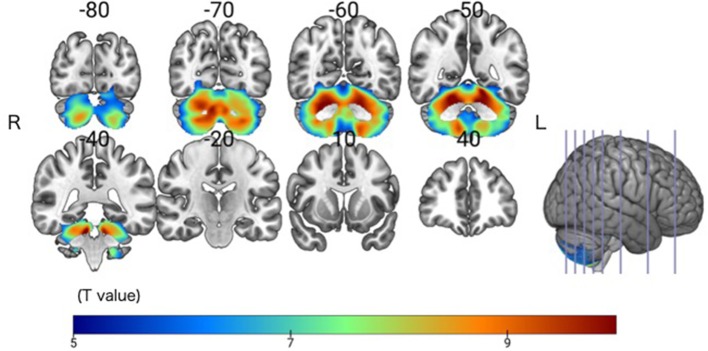
Significant GMV atrophy in the SCD group compared with the HC group. Coronal brain sections. Individuals with SCD had smaller GMVs compared with the HC group in widespread regions of the cerebellum. FWE corrected *p* < 0.05.

The SCD group had significantly smaller GMV in the cerebellar right hemispheric Lobule IV, V, VI, VIII, and IX, and in the left hemispheric Lobule IV, V, VI, VIII, and IX, and Crus I and II (Peak-level threshold of FWE corrected *p* < 0.05) (Figure 2, Table 3).

**Table 3:** Clusters of significant GM atrophy in the SCD group relative to the HC group.

**Cluster voxel no**.	**Region**	**Side**	**T score**	**MNI coordinates, mm**		
				***x***	***y***	***z***
36124	Cerebellum hemisphere (lobule IV-V)	R	9.37	18	−50	−24
	Cerebellum hemisphere (lobule VI)	R	9.86	22	−62	−33
	Cerebellum hemisphere (lobule VIII)	R	9.36	9	−66	−33
	Cerebellum hemisphere (lobule IX)	R	8.29	10	−50	−54
	Cerebellum hemisphere (lobule IV-V)	L	9.20	−15	−51	−15
	Cerebellum hemisphere (lobule VI)	L	9.20	−16	−56	−27
	Cerebellum hemisphere (Crus I)	L	7.18	−33	−60	−46
	Cerebellum hemisphere (Crus II)	L	8.29	−26	−72	−46
	Cerebellum hemisphere (lobule VIII)	L	8.98	−8	−66	−33
	Cerebellum hemisphere (lobule IX)	L	7.05	−12	−50	−51

*All T-scores were significant with the threshold of p <0.05 (FWE-corrected). MNI Coordinates, Montreal Neurologic Institute coordinates*.

### Correlations Between Adaptive Learning Ability Parameters and GMV in the SCD Group

**Figure 3:**
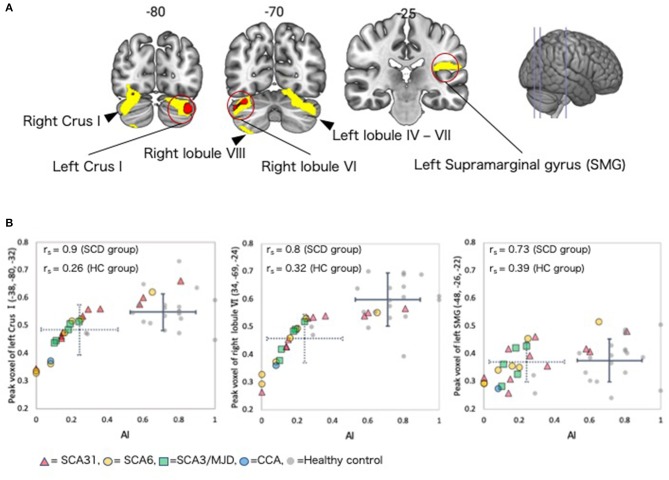
Regions with correlations between GMV and *AI* in the SCD group. **(A)** Coronal sections showing regions in which gray matter was significantly correlated with *AI*. Red color = peak-level *p* < 0.05 (FWE corrected). Yellow color, cluster-level *p* < 0.05 (FWE corrected). **(B)** Scatter plot depicting the correlation between regional GMV at the peak voxel and *AI*. The dotted crossed line shows the mean and standard deviation (SD) of the *AI* and peak voxel values in the SCD group. The solid crossed line shows the mean and SD of the *AI* and peak voxel values in the HC group. r_s_, Spearman's rank order correlation coefficients; SMG, supramarginal gyrus.

In the SCD group, a whole-brain multiple regression analysis indicated that lower *AI* scores were associated with smaller GMV in the right cerebellar hemispheric lobule VI and left cerebellar Crus I (Peak-level threshold of FWE corrected *p* < 0.05, Figure 3A, Table 4). Additionally, a complementary multiple regression analysis identified the left supramarginal gyrus, the right cerebellar hemisphere (Crus I and lobule VI–VIII) and the left cerebellar hemisphere (lobule IV–VI and Crus I) showing non-negligible association between GM atrophy and reduced *AI* in the SCD group (Cluster-level threshold of FWE corrected *P* < 0.05) (Figure 3A, Table 5). We did not observe any clear differences among individuals with different types of SCA in terms of the correlation pattern between AI score and cerebellar or parietal GMV (Figure 3B). There was no correlation between AI and GMV within the HC group at both thresholds. The τ*_log* values were not significantly correlated with GMV in either the SCD group or the HC group even at the liberal threshold.

**Table 4:** Clusters with significant correlations between GM volume and *AI* in the SCD group (peak-level corrected).

**Cluster voxel no**.	**Region**	**Side**	**T score**	**MNI coordinates, mm**		
				***x***	***y***	***z***
78	Cerebellum hemisphere (lobule VI)	R	7.08	34	−69	−24
51	Cerebellum hemisphere (Crus I)	L	6.68	−38	−80	−32

*All T-scores were significant with a threshold of p <0.05 (FWE-corrected). MNI Coordinates, Montreal Neurologic Institute coordinates*.

**Table 5:** Clusters with significant correlations between GM volume and *AI* in the SCD group (cluster-level corrected).

**Cluster voxel no**.	**Region**	**Side**	**P_**FWE-corr**_**	**MNI coordinates, mm**		
				***x***	***y***	***z***
1403	Supramarginal gyrus	L	0.005	−48	−26	22
4189	Cerebellum hemisphere (lobule VI)	R	0.0001	34	−69	−24
	Cerebellum hemisphere (Crus I)	R		36	−84	−38
		R		48	−69	−32
		R		26	−90	−32
		R		18	−92	−27
		R		38	−80	−28
1012	Cerebellum hemisphere (lobule VIII)	R	0.02	42	−63	−56
	Cerebellum hemisphere (lobule VII)	R		38	−70	−58
6595	Cerebellum hemisphere (lobule IV–V)	L	0.0001	−16	−36	−16
		L		−24	−32	−26
	Cerebellum hemisphere (lobule VI)	L		−22	−76	−18
	Cerebellum hemisphere (Crus I)	L		−46	−62	−26

*All P-value were significant at a cluster-level threshold of p <0.05 (FWE-corrected, uncorrected p <0.001 at voxel-level). MNI Coordinates, Montreal Neurologic Institute coordinates*.

## Discussion

The main finding of the present study was that reduction *AI* was correlated with GM atrophy in the cerebellum and parietal cortex in the SCD group, pooling across SCA types and CCA. We observed three main types of SCA amongst our participants. SCA6 is an autosomal dominant type of cerebellar ataxia, and is considered to be a “pure” type of cerebellar ataxia (32). SCA31 is a type of elderly-onset, pure cerebellar ataxia that is unique in Japan (38). In both SCA6 and SCA31, Purkinje cells, which are the only projecting neurons in the cerebellar cortex, are predominantly affected (26, 27). In SCA3/MJD, degenerative processes involve widely distributed parts of the brain, including the cerebral cortex, basal ganglia, and pontomedullary systems, in addition to the cerebellum (22, 24).

The studied SCA subtypes were consistent with those included in the previous behavioral study by Hashimoto et al. (20). In their study, 17% of the SCD patients (12 out of 70) showed an *AI* of 0; consistently, 13% of the SCD patients (3 out of 23) showed an *AI* of 0 here. This finding of apparently “no adaptation at all (*AI* = 0)” reflects the design of the equation computing *AI* in which 0 in any one of the parameters (a, b, and c) results in *AI* of 0, emphasizing a subtle adaptation failure. Moreover, we replicated significant correlation between SARA and *AI*. Overall, these findings validated the clinical usefulness of *AI* for assessing patients with SCD. The failure to replicate the relationship between *AI* and disease duration suggests that the current cohort contained various types of SCDs (32, 38).

Our VBM results showed that *AI* score was correlated with GMV in the right cerebellar hemisphere (Crus I and lobule VI–VIII) and the left cerebellar hemisphere (lobule IV–VI and Crus I) in the SCD group. Particularly, the right lobule VI and the left Crus I showed most robust correlation. The supporting evidence is available from three perspectives, described below. First, these cerebellar regions in which GMV was correlated with *AI* score are consistent with the correlates of prism adaptation in previous human and non-human primate studies. Lesion (39) and pharmacological inactivation (40) studies in non-human primates have shown that cerebellar hemisphere lobules VII, VIII, IX, and the dentate nucleus were involved in prism adaptation during a reaching task. Furthermore, human lesion studies have suggested that cerebellar hemispheres IV, V, and VI are involved in adaptation during the hand-reaching task (41, 42).

Second, the right lobule VI and left Crus I have been found to be the correlates of eye-hand coordination required for prism adaptation. The lobule VI likely corresponds to hand representations in the cerebellum, which are somatotopically organized (43), and an MRI study indicated that the right cerebellar hemisphere IV–VI was active during a tapping task involving the right upper limb (44). In our study, all participants performed the task with their right hand. This explains our finding with respect to GMV in lobule VI in the right cerebellar hemisphere. In addition, the left cerebellum has been found to be active in spatial cognition task (44). This explains our finding regarding GMV in the Crus 1 in the left hemisphere.

Third, although *AI* is a composite score summarizing several aspects of behavior during prism adaption, AI primarily reflects a late phase of adaptation learning in which the cerebellar VI and VII may play a critical role. The cerebellum (e.g., lobule VI) constitutes a semi-closed loop with the primary motor area (M1), thereby providing neural circuits for adaptive learning using reaching error as a training signal. This semi-closed loop is expected to feed the efference copy to the cerebellar internal model, which also receives error-related signals from the climbing fibers, and the dentate nucleus projects back to the M1 (9). As the prism adaptation task poses learning of new voluntary visuomotor behavior, it would be necessary for participants to reconstruct cerebellar internal models for reaching (45). A unit-recording study in monkeys indicated that complex spikes from the Purkinje cells in cerebellar hemispheric lobules IV–VI encode hand-reaching error signals (46). An fMRI study using prism adaptation showed that cerebellar hemisphere lobules VIII and IX, as well as the dentate nucleus, were active in the early phase of adaptation (47) while cerebellar lobules IV and VI were active in the late phase of adaptation (41, 47, 48). To calculate *AI*, we used the 10 last trials in the PRISM session. Thus, our results regarding *AI* may reflect activity associated with the late phase of adaptation. On the basis of their human fMRI study, Diedrichsen et al. suggested that hand-reaching error signals are encoded in cerebellar lobules V, VI, and VIII, as well as in the dentate nucleus (49). In addition, they found that the cerebellar hemisphere VI and the left cerebellar hemisphere VII (Crus I) were active during the prism adaptation task. Their results are in accordance with the VBM analysis results of the present study.

A complementary analysis (uncorrected *P* < 0.001 with cluster-level corrected *P* < 0.05) showed correlation between *AI* and GMV of the left supramarginal gyrus. In previous fMRI studies, the inferior parietal lobule including the supramarginal gyrus has been shown active in prism adaptation tasks (48, 50, 51). In the present study, the SCD group did not show clear atrophy in the parietal cortex (see right panel of Figure 3B), making it unlikely to interpret this finding as a direct effect of neural degeneration. Alternatively, it is possible that the decrease in *AI* is influenced by aging since a previous study showed that *AI* was significantly reduced in healthy controls over 70 years of age (20). Also, GMV of the cerebellum and cerebral cortex (including parietal lobe) decreases in association with aging (52, 53). However, the effect of aging is unlikely explanation because age was used as a covariate in the multiple regression analysis. Although the direct evidence is yet to be available, we speculate that the finding in the parietal cortex could result from remote effects of cerebellar degeneration onto the functional organization of the supramarginal gyrus, through the cortico-cerebellar circuits. To test this hypothesis in the future, we should analyze functional connectivity MRI data (54).

We also analyzed τ*_log* from the prism adaptation data. Although we found a statistically significant difference between the groups, we did not find a significant correlation between τ*_log* and cerebellar GMV in the VBM analysis. Researchers have proposed that prism adaptation consists of two phases (55, 56). The early phase encompasses the time required to consciously detect errors immediately after wearing the prism, and the late phase is characterized by recalibration of spatial maps. An event-related fMRI study showed that the posterior parietal cortex was activated during error detection in the early phase (48). Because it is mainly calculated using data from the early phase, τ may not reflect a purely cerebellar function.

## Limitations

We pooled all of the participants with SCD in the multiple regression analysis because of the small number of participants with each SCA type. In future studies, it will be necessary to recruit more patients with each SCA type, and then conduct a multiple regression analysis taking the genotype into account. In this study, we investigated the relationship between localized cerebellar regions and motor learning ability (*AI*) in SCD. Previous resting-state fMRI studies in healthy people have suggested that the cerebellum is functionally connected to most cerebral cortical areas (57). In future research, network analyses using functional and effective connectivity will be important to fully understand the roles of the cerebellum as a network component implicated in prism adaptation problems in patients with SCD. Finally, in order to show that *AI* is useful as a clinical index, it is necessary to verify its reliability and validity test.

## Conclusions

We examined the neural correlates of parameters measured via a prism adaptation task (*AI* and τ*_log*) in SCD using VBM. We discovered that *AI*, a recently proposed composite score to summarize several aspects of motor adaption, was associated with GMV loss in cerebellar sub-sectors and the inferior parietal lobule in patients with SCD. The brain regions found here for the correlates of prism adaptation were consistent with those from the previous studies (39–42, 46–49, 58). A novel point of our study is that we found a correlation between decreased sensorimotor adaptation and decreased GMV not only in the cerebellum but also in the parietal lobe in SCD patients. This finding should be further tested in the future from the viewpoint of cortico-cerebellar circuits. SCD is a progressive disease, which is difficult to treat at this point. For better management of the SCD patients, the development of biomarkers that can reflect the degree of disease progression is important. We believe that this study will contribute to development of biomarkers for SCD.

## Data Availability Statement

Deidentified participant data will be shared, as well as the study protocol and statistical analyses, upon reasonable requests.

## Ethics Statement

The studies involving human participants were reviewed and approved by the Bioethics Committee of National Center of Neurology and Psychiatry. The patients/participants provided their written informed consent to participate in this study.

## Author Contributions

KB and THo conceived and designed the study, and acquired the data. KB analyzed the data and drafted the manuscript. KB and THa interpreted the data. KI, YT, and HM critically reviewed the manuscript.

### Conflict of Interest

The authors declare that the research was conducted in the absence of any commercial or financial relationships that could be construed as a potential conflict of interest.
